# Mefloquine—An Aminoalcohol with Promising Antischistosomal Properties in Mice

**DOI:** 10.1371/journal.pntd.0000350

**Published:** 2009-01-06

**Authors:** Jennifer Keiser, Jacques Chollet, Shu-Hua Xiao, Jin-Yan Mei, Pei-Ying Jiao, Jürg Utzinger, Marcel Tanner

**Affiliations:** 1 Department of Medical Parasitology and Infection Biology, Swiss Tropical Institute, Basel, Switzerland; 2 National Institute of Parasitic Diseases, Chinese Center for Disease Control and Prevention, Shanghai, People's Republic of China; 3 Department of Public Health and Epidemiology, Swiss Tropical Institute, Basel, Switzerland; Rush University Medical Center, United States of America

## Abstract

**Background:**

The treatment and control of schistosomiasis, an often neglected tropical disease that exacerbates poverty, depends on a single drug, praziquantel. The large-scale use of praziquantel might select for drug-resistant parasites, hence there is a need to develop new antischistosomal compounds. Here, we report that the antimalarial drug mefloquine possesses promising antischistosomal properties in mice.

**Methodology/Principal Findings:**

A single dose of mefloquine (200 or 400 mg/kg) administered orally to mice infected with adult *Schistosoma mansoni* or adult *S. japonicum* resulted in high or complete total and female worm burden reductions (72.3%–100%). Importantly, high worm burden reductions were also observed for young developing stages of *S. mansoni* and *S. japonicum* harbored in the mouse. Both mefloquine erythro-enantiomers resulted in high and comparable total and female worm burden reductions when given to mice with either a sub-patent or patent *S. mansoni* infection.

**Conclusions/Significance:**

Our findings hold promise for the development of a novel antischistosomal drug based on an aminoalcohol functionality. Further *in vitro* and *in vivo* studies have been launched to elucidate the possible mechanism of action and to study the effect of mefloquine on *S. haematobium* and other trematodes. It will be interesting to investigate whether mefloquine, which is widely and effectively used for the treatment of malaria, has an impact on schistosomiasis in areas where both malaria and schistosomiasis co-exist.

## Author Summary

Schistosomiasis is a chronic and debilitating disease that occurs in tropical and subtropical areas. The disease is caused by an infection with a parasitic worm and affects over 200 million people. The treatment and control of schistosomiasis relies on a single drug, praziquantel. This drug is increasingly used, and hence there is mounting concern about the development of resistance to praziquantel. Here we report that mefloquine, a marketed drug for prophylaxis and treatment of malaria, shows promising antischistosomal properties in laboratory studies with mice. When mefloquine was orally administered at a single dose of 200 or 400 mg/kg to mice infected with young or adult stages of the parasitic worm *Schistosoma mansoni* or *S. japonicum*, we found very high worm burden reductions. We also found high worm burden reductions when mefloquine enantiomers were given to mice infected with adult *S. mansoni*. Further studies are needed because our results might be of public health relevance. Indeed, mefloquine is widely used for prophylaxis and treatment of malaria, often in areas where both malaria and schistosomiasis co-exist. In such areas, it might be possible that the use of mefloquine against malaria reduces the burden of schistosomiasis.

## Introduction

Schistosomiasis is a chronic and debilitating disease that exacerbates poverty [1],[2]. Although close to 800 million individuals are at risk of contracting the disease and over 200 million people are thought to be infected, schistosomiasis is often neglected [3],[4]. The causative agent is a digenetic trematode of the genus *Schistosoma*. The three main species parasitizing humans are *S. haematobium*, *S. japonicum*, and *S. mansoni*. Morbidity due to schistosomiasis includes hepatic and intestinal fibrosis (*S. mansoni* and *S. japonicum*), and ureteric and bladder fibrosis and calcification of the genitourinary tract (*S. haematobium*) [5]. The global burden of schistosomiasis has been estimated at 1.7 to 4.5 million disability-adjusted life years (DALYs), but even the higher estimate might be an underestimation of the true burden [2],[6],[7].

The treatment and control of schistosomiasis virtually relies on a single drug, praziquantel. The pressing need to develop new antischistosomal compounds has been emphasized [8]–[11], particularly in view of blanket application of praziquantel within the frame of ‘preventive chemotherapy’ [12], a strategy that might select for drug-resistant parasites. Additionally, there is an important deficiency in the therapeutic profile of praziquantel. The drug targets the adult worm, but has only minor activity against the young developing stages (i.e., schistosomula); hence, retreatment is necessary to kill those parasites that have since matured. There is no dedicated drug discovery and development program pursued for schistosomiasis, either by the pharmaceutical industry or through public-private partnerships. However, despite the paucity of a concerted effort to develop novel antischistosomal drugs, a number of compounds with promising antischistosomal properties have been identified by academia, such as the synthetic trioxolanes [13], the cysteine protease inhibitor K11777 [14], alkylaminoalkanethiosulfuric acids [15], praziquantel analogs [16] and, most recently, the oxadiazoles [10]. Nonetheless, to develop a new antischistosomal drug from lead drug candidates will take at least another decade. Underlying reasons are the scarce resources available for schistosomiasis and other neglected tropical diseases and the high failure rates of compounds during preclinical and clinical testing [17].

Interestingly, the artemisinins (e.g., artemether and artesunate), which are essential components of malaria treatment and control [18], also possess antischistosomal properties [19],[20]. Detailed *in vivo* studies revealed that schistosomula are particularly susceptible to the artemisinins, whereas moderate worm burden reductions are still apparent for adult worms [21]. A number of clinical trials carried out in different African settings confirmed that both artemether and artesunate have an effect against patent infections with *S. haematobium* and *S. mansoni* [20],[22]. Artemisinin-based combination therapies (ACTs) have been adopted as first-line drugs for uncomplicated *Plasmodium falciparum* malaria in most malaria-endemic countries as a strategy to avoid the selection of parasite drug resistance [18]. Since large parts of Africa are co-endemic for malaria and schistosomiasis [22] and both *Plasmodium* and *Schistosoma* parasites degrade hemoglobin, a putative target for several antimalarial drugs, we were motivated to test other antimalarials that are commonly employed in combination with an artemisinin derivative for their potential antischistosomal activities. We then followed up on the promising *in vivo* activity of mefloquine, first to elucidate the dose-response relationships of single-dose mefloquine against juvenile and adult *S. mansoni* and *S. japonicum* and, second, to assess the stage-specific susceptibility of both parasites to mefloquine. Finally, we evaluated the activity of mefloquine (+) and (−) erythro enantiomers against different developmental stages of *S. mansoni.*

## Materials and Methods

### Drugs

Halofantrine, mefloquine HCl, and mefloquine enantiomers were obtained from Hoffmann La Roche (Basel, Switzerland); pyronaridine was provided by the National Institute of Parasitic Diseases, Chinese Center for Disease Control and Prevention (Shanghai, China); and pyrimethamine, sulfadoxine, and sulfamethoxypyrazine were obtained from Dafra Pharma (Turnhout, Belgium). For the *in vivo* studies with *S. mansoni*, lumefantrine was provided by the Novartis Institute for Biomedical Research (Basel, Switzerland); amodiaquine, chloroquine, and quinine were purchased from Sigma (Buchs, Switzerland); and atovaquone was purchased in a local Swiss pharmacy (Wellvone®). For the *in vivo* studies with *S. japonicum*, lumefantrine was obtained from Kunming Pharmaceutical Corporation (Kunming, China); and amodiaquine, atovaquone, chloroquine, and halofantrine were provided by the National Institute of Parasitic Diseases, Chinese Center for Disease Control and Prevention (Shanghai, China).

All drugs were prepared as suspensions in 7% (v/v) Tween 80 and 3% (v/v) ethanol before oral administration to mice (10 ml/kg).

### Animals and parasites

Experiments with *S. mansoni* (Liberian strain) were carried out at the Swiss Tropical Institute (Basel, Switzerland), in accordance with Swiss national and cantonal regulations on animal welfare (permission no. 1731). Female mice (NMRI strain, *n* = 290, weight ∼20–22 g) were purchased from RCC (Itingen, Switzerland). Mice were kept under environmentally-controlled conditions (temperature ∼25°C; humidity ∼70%; 12-hour light and 12-hour dark cycle) and acclimatized for one week before infection. The animals had free access to water and rodent diet.

The experiments with *S. japonicum* (Anhui strain) were undertaken at the National Institute of Parasitic Diseases, Chinese Center for Disease Control and Prevention. Male mice (Kunming strain, *n* = 125, weight ∼20–22 g) were purchased from Shanghai Experimental Animal Center of the Chinese Academy of Sciences (Shanghai, China).

Cercariae of *S. mansoni* and *S. japonicum* were obtained from infected intermediate host snails in our laboratories as described previously [13].

### *In vivo* studies with *S. mansoni*

Each mouse was infected subcutaneously with ∼80 *S. mansoni* cercariae. Twenty-one days (pre-patent infection) and 49 days (patent infection) after the experimental infection, groups of 3–5 mice were treated orally with the drugs to be tested at single oral doses (25–400 mg/kg). To study the stage-specific susceptibility of *S. mansoni*, mice were treated with a single 400 mg/kg oral dose of mefloquine either 2 days or 1 day before infection, 3 hours after infection or at days 7, 14, 21, 28, 35, 42, and 49 post-infection. For the hepatic shift experiments groups of mice were treated with 400 mg/kg mefloquine 49 days post-infection, and the worm distribution was analyzed on days 1, 3, 7 and 14 post-treatment. Results of the hepatic shift experiments are used as an additional criterion for evaluating antischistosomal drugs since this test shows how quickly the forced dislodgement of worms occurs [23]. For each experiment, groups of 5–10 untreated mice served as controls.

At 21 days post-treatment, animals were killed by the CO_2_ method and dissected, and worms were sexed and counted as described elsewhere [13]. *In vivo* experiments with *S. mansoni* were carried out in duplicates. The results from the second set of experiment are summarized in Supporting Tables S1, S2, and S3.

### *In vivo* studies with *S. japonicum*

Mice were infected percutaneously with ∼40 *S. japonicum* cercariae each. To investigate the dose-response relationship of mefloquine against juvenile and adult *S. japonicum*, single 25–400 mg/kg oral doses were given to mice 14 days (pre-patent infection) and 35 days (patent infection) post-infection. To assess the efficacy of mefloquine against different stages of *S. japonicum*, mice were treated with a single oral dose of 400 mg/kg mefloquine 2 days or 1 day before infection, 3 hours after infection, and at days 3, 7, 14, 21, 28 and 35 post-infection. In each experiment, infected but untreated mice served as controls.

Twenty-one days post-treatment, mice were killed and the worms recovered from the hepatic and portomesenteric veins by the perfusion technique [24]. To study the hepatic shift in adult *S. japonicum*, groups of mice were treated with 400 mg/kg mefloquine 35 days post-infection, and the worm distribution was analyzed on days 1, 3, 7, and 14 post-treatment.

### Statistical analysis

For statistical analysis we used version 2.4.5 of the Statsdirect statistical software package (Cheshire, United Kingdom). The Kruskal-Wallis (KW) test, which compares the medians of the responses between the treatment and control groups, was used. A difference in median was considered to be significant at a significance level of 5%.

## Results

### Effect of selected antimalarials on *S. mansoni* harbored in mice

**Figure 1:**
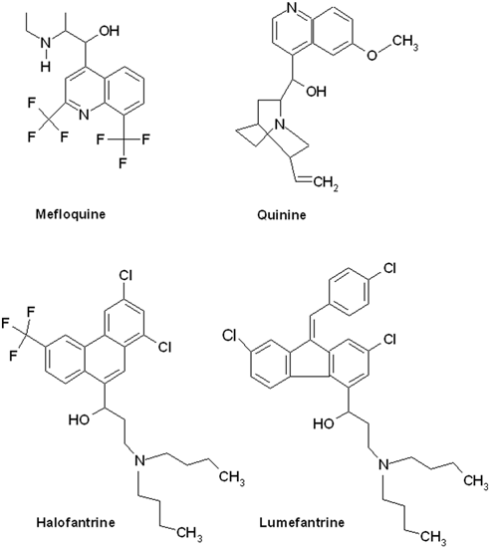
Chemical structures of mefloquine, quinine, halofantrine, and lumefantrine.

The *in vivo* antischistosomal efficacy of 11 antimalarial drugs is summarized in Tables 1 and 2. Drugs were administered orally at a single dose of 400 mg/kg to mice harboring adult *S. mansoni*, and worm burden reductions, including changes in worm distributions, were assessed. Amodiaquine, atovaquone, lumefantrine, pyrimethamine, pyronaridine, sulfadoxine, and sulfamethoxypyrazine showed no antischistosomal activity. Quinine and halofantrine resulted in total and female worm burden reductions ranging between 51.7% and 74.9% and changes in the worm distribution. The highest activity (total and female worm burden reduction of 77.3% and 100%, respectively) was observed with a single dose of mefloquine (400 mg/kg), which was statistically significant (*p<*0.05). The chemical structures of the four aminoalcohols quinine, halofantrine, lumefantrine, and mefloquine are shown in Figure 1.

**Table 1:** Effect on worm burden of a single oral dose of seven selected antimalarials administered to mice harboring a 49-day-old adult *S. mansoni* infection, stratified by sex and worm distribution.

Group	Dose (mg/kg)	No. of mice investigated	No. of mice cured	Mean number of worms (SD)					Worm burden reduction (%)	
				Liver	Mesenteric veins	Total	Males	Females	Total	Female
Control	–	5	–	1.4 (0.9)	24.6 (8.4)	26.0 (7.8)	14.0 (3.8)	12.0 (4.0)	–	–
Amodiaquine	400	3^a^	0	1.5 (0.7)	41.5 (6.4)	43.0 (5.7)	22.5 (2.1)	20.5 (3.5)	0	0
Atovaquone	400	3	0	0.3 (0.6)	>30	>30	n.d	n.d	0	0
Pyrimethamine	100	3^b^	0	0	39.0	40.0	21.0	19.0	0	0
Pyronaridine	400	3	0	2.7 (2.1)	24.0 (11.3)	26.7 (9.3)	15.3 (2.9)	11.3 (6.4)	0	5.8
Sulfadoxine	400	3^a^	0	0.7 (0.6)	10. 7 (10.5)	28.5 (21.9)	14.5 (10.6)	14.5 (10.6)	0	0
Sulfamethoxypyrazine	400	3^c^	0	8.0 (0)	34.5 (7.8)	42.5 (7.8)	27.5 (3.5)	15.0 (4.2)	0	0
Control	–	10	–	4.3 (3.3)	30.2 (8.2)	34.3 (8.4)	20.1 (5.3)	14.2 (4.5)	–	–
Chloroquine	400	5^d^	0	2.7 (1.5)	27.3 (4.7)	30.0 (4.4)	17.3 (3.5)	12.7 (2.1)	11.7	0

SD, standard deviation.

a One mouse died on day 27 post-treatment.

b Two mice died on days 1 and 2 post-treatment.

c One mouse died on day 22 post-treatment.

d Two mice died on days 27 and 30 post-treatment.

**Table 2:** Effect on worm burden of a single 400 mg/kg oral dose of four selected aminomethanol antimalarials administered to mice harboring a 49-day-old adult *S. mansoni* infection, stratified by sex and worm distribution.

Group	No. of mice investigated	No. of mice cured	Mean number of worms (SD)					Total worm burden reduction (%)	KW	*p*-value	Female worm burden reduction (%)	KW	*p*-value
			Liver	Mesenteric veins	Total	Males	Females						
Control	10	–	4.3 (3.3)	30.2 (8.2)	34.3 (8.4)	20.1 (5.3)	14.2 (4.5)	–	–	–	–	–	–
Lumefantrine	5^a^	0	2.3 (1.5)	28.7 (4.7)	35.3 (4.0)	18.0 (1.0)	14.0 (3.5)	10.5	0.18	0.671	1.4	0.02	0.864
Mefloquine	4	0	7.0 (3.6)	0.8 (1.5)	7.8 (5.0)	7.8 (5.0)	0	77.3	8.02	0.005	100	9.77	0.002
Quinine	5	0	2.6 (0.9)	13.0 (8.2)	15.6 (7.4)	10.2 (2.9)	5.4 (4.6)	54.7	7.97	0.005	62.0	5.75	0.016
Control	10	–	5.5 (3.1)	38.9 (15.3)	46.6 (14.5)	27.3 (9.3)	18.3 (7.5)	–	–	–	–	–	–
Halofantrine	4	0	16.5 (11.3)	0.5 (1.0)	22.5 (14.2)	16.8 (11.3)	7.8 (3.4)	51.7	4.22	0.039	57.4	6.88	0.009

KW, Kruskal-Wallis; SD, standard deviation.

a Two mice died on days 17 and 34 post-treatment.

### Dose-response relationships of mefloquine against juvenile and adult *S. mansoni*

In view of the promising antischistosomal activity of mefloquine, its properties were further characterized, with an emphasis on dose-response relationships in juvenile (21-day-old) and adult (49-day-old) *S. mansoni* harbored in mice (Table 3, Table S1). In the juvenile infection model, total and female worm burden reductions of 94.2–100% were achieved with a single-dose oral regimen (100 mg/kg and above). At a dose of 50 mg/kg, the total and female worm burden reductions were 30.8% and 38.3%, respectively. At the lowest dose investigated (25 mg/kg) mefloquine showed no effect on juvenile *S. mansoni* in the mouse. The difference in total and female worm burdens between mice infected with 21-day-old juvenile *S. mansoni* that were treated (25–400 mg/kg) and those mice left untreated was highly significant (KW = 9.51, *p* = 0.002 and KW = 8.16, *p* = 0.004, respectively).

**Table 3:** Dose-response relationship of mefloquine administered to mice harboring a 21-day-old juvenile and a 49-day-old adult *S. mansoni* infection.

Stage of infection	Dosage (mg/kg)	No. of mice investigated	No. of mice cured	Mean number of worms (SD)					Total worm burden reduction (%)	KW	*p*-value	Female worm burden reduction (%)	KW	*p*-value
				Liver	Mesenteric veins	Total	Males	Females						
Juvenile	–	10	–	4.9 (3.3)	36.6 (7.7)	41.5 (8.0)	23.0 (4.7)	18.5 (4.1)	–			–		
	25	5	0	3.5 (1.6)	46.6 (11.0)	50.2 (10.8)	26.8 (7.1)	23.8 (4.6)	0	9.51	0.002	0	8.16	0.004
	50	5	0	2.2 (1.9)	23.4 (7.5)	25.6 (9.1)	12.8 (4.5)	12.8 (4.9)	38.3			30.8		
	100	5	2	1.2 (1.3)	1.2 (1.1)	2.4 (2.3)	1.4 (1.3)	1.0 (1.0)	94.2			94.6		
	200	5	1	1.2 (0.8)	1.2 (1.8)	2.4 (2.3)	1.6 (1.7)	0.8 (0.8)	94.2			95.7		
	400	5	1	1.0 (0.7)	0	1.0 (0.7)	1.0 (0.7)	0	97.6			100		
Adult	–	10	–	4.3 (3.3)	30.2 (8.2)	34.3 (8.4)	20.1 (5.3)	14.2 (4.5)	–			–		
	25	4	0	2.3 (3.3)	27.5 (5.3)	29.8 (5.3)	16.3 (3.6)	13.5 (1.7)	13.1	12.49	<0.001	4.9	9.46	0.002
	50	5	0	3.0 (2.1)	19.6 (8.6)	22.6 (6.9)	13.0 (3.3)	9.6 (3.9)	34.3			32.4		
	100	5	0	8.8 (4.4)	9.8 (4.5)	18.6 (2.5)	12.4 (2.4)	6.2 (2.3)	45.8			56.3		
	200	5^a^	0	5.5 (2.6)	4.0 (5.0)	9.5 (4.8)	8.5 (3.9)	1.0 (2.0)	72.3			93.0		
	400	5^b^	0	7.0 (3.6)	0.8 (1.5)	7.8 (5.0)	7.8 (5.0)	0	77.3			100		

Worm burden is stratified by sex and worm distribution.

KW, Kruskal Wallis test; SD, standard deviation.

a one mouse died 13 days post-treatment.

b one mouse died 11 days post-treatment.

Oral administration of mefloquine at a single dose (200 mg/kg and 400 mg/kg) to mice infected with adult *S. mansoni* resulted in total and female worm burden reductions of 72.3–100%. No or only moderate total and female worm burden reductions (4.9–56.3%) were achieved with a single dose of 25, 50 or 100 mg/kg mefloquine. There was a highly significant difference between the total and female worm burden of mefloquine-treated mice (25–400 mg/kg) and control mice in the adult infection model (KW = 12.49, *p*<0.001 and KW = 9.46, *p* = 0.002, respectively).

### Stage-specific susceptibility *S. mansoni*

Tables 4 and Table S2 summarizes the activity of mefloquine when given 2 days or 1 day before infection, shortly after infection (3 hours post-infection) and until 49 days post-infection. These experiments were carried out with a single oral dose of 400 mg/kg mefloquine as this dose achieved the highest reductions in worm burden against juvenile and adult *S. mansoni*. A single oral dose of mefloquine was highly active against mice harboring either a 7-, 14-, 21-, 28-, 35-, 42-, or 49-day-old *S. mansoni* infection (total and female worm burden reductions ranged between 83.9% and 100%). Mefloquine administration to mice 2 days or 1 day before infection or 3 hours after infection showed moderate total and female worm burden reductions (35.9–46.5%). Regardless of the timing of mefloquine administration, i.e., shortly before infection or administration when mice were infected with juvenile or adult *S. mansoni*, total and female worm burden reductions were highly significant (*p*<0.001).

**Table 4:** Stage-specificity of a single 400 mg/kg oral dose mefloquine administered to mice infected with *S. mansoni*, stratified by sex and worm distribution.

Drug administration		No. of mice investigated	No. of mice cured	Mean number of worms (SD)					Total worm burden reduction (%)	KW	*p*-value	Female worm burden reduction (%)	KW	*p*-value
				Liver	Mesenteric veins	Total	Males	Females						
Pre-infection	Control	10	–	4.1 (1.8)	31.6 (6.4)	35.9 (6.3)	20.2 (3.3)	15.7 (3.5)	–	22.98	<0.001		23.27	<0.001
	Day -2	5	0	5.4 (5.2)	17.6 (6.1)	23.0 (5.6)	14.6 (4.9)	8.4 (2.9)	35.9			46.5		
	Day -1	5^a^	0	4.0 (2.5)	16.5 (8.2)	20.5 (8.3)	12.0 (4.3)	8.5 (4.2)	42.9			45.9		
Post-infection	Day 0	10^a^	0	2.6 (3.5)	17.9 (8.7)	20.4 (8.5)	11.9 (4.8)	8.6 (4.7)	43.2			45.2		
	Day 7	5^a^	0	1.5 (2.4)	4.3 (3.1)	5.8 (0.9)	3.5 (1.0)	2.3 (1.7)	83.9			85.4		
	Day 14	5	1	1.0 (1.2)	2.0 (2.0)	3.0 (2.7)	1.8 (1.9)	1.2 (1.1)	91.6			92.4		
	Day 21	5^a^	4	0	0	0	0	0	100			100		
	Day 28	5	4	0.4 (0.9)	0	0.4 (0.9)	0.4 (0.9)	0	98.9			100		
	Day 35	5	3	0.6 (0.9)	0	0.6 (0.9)	0.6 (0.9)	0	98.3			100		
	Day 42	5	0	2.2 (2.3)	0	2.2 (2.3)	2.2 (2.3)	0	93.8			100		
	Day 49	5	0	2.4 (0.9)	0	2.4 (0.9)	2.4 (0.9)	0	93.3			100		

KW, Kruskal-Wallis test; SD, standard deviation.

a One mouse died several days post-treatment.

### Activity of mefloquine erythro-enantiomers against juvenile and adult *S. mansoni*

Both (−)(11*S*, 2′*R*) and (+)(11*R*,2′*S*) erythro-enantiomers of mefloquine resulted in high and comparable total and female worm burden reductions (*p*<0.001) when given to mice infected with either juvenile or adult stages of *S. mansoni*. At a single dose of 100 mg/kg and above, mefloquine (−)(11*S*, 2′*R*) achieved worm burden reductions of 57.1% to 100% in mice harboring 21-day-old juvenile and 49-day-old adult *S. mansoni.* At a single dose of 50 mg/kg, total worm burden reductions of 8.7% to 19.9% were observed in the juvenile and adult infection model, respectively (Table 5).

**Table 5:** Effect of mefloquine (+) and (−) enantiomer administered to mice infected with either juvenile or adult *S. mansoni.*

Enantiomer tested	Stage of infection	Dosage (mg/kg)	No. of mice cured/investigated	Mean number of worms (SD)					Total worm burden reduction (%)	KW	*P*-value	Female worm burden reduction (%)	KW	*p*-value
				Liver	Mesenteric veins	Total	Males	Females						
	Control	–	-/10	3.9 (3.2)	28.3 (7.3)	32.2 (9.0)	17.6 (5.0)	14.6 (4.4)	–			-		
(+)(11*R*,2′*S*) RO13-7224	Juvenile	50	0/5	1.6 (1.1)	14.8 (5.8)	16.4 (5.8)	8.4 (3.8)	8.0 (2.8)	49.1	17.69	<0.001	45.2	16.70	<0.001
		100	0/5	1.4 (0.9)	1.4 (0.9)	2.8 (0.8)	1.8 (1.1)	1.0 (0.7)	91.3			93.1		
		200	1/5	0.8 (0.4)	0.2 (0.4)	1.0 (0.7)	0.8 (0.8)	0.2 (0.4)	96.9			98.6		
		400	3/5^a^	0	0	0	0	0	100			100		
	Adult	50	0/5	5.4 (1.8)	14.8 (4.3)	20.6 (4.1)	12.0 (3.2)	8.2 (1.5)	36.0	16.25	<0.001	43.8	18.56	<0.001
		100	0/5	10.4 (4.1)	0.8 (1.3)	11.6 (5.3)	9.8 (5.1)	1.8 (0.8)	64.0			87.7		
		200	0/5	4.2 (2.2)	1.0 (1.2)	5.2 (2.5)	4.6 (2.6)	0.6 (1.3)	83.8			95.9		
		400	2/5	2.2 (2.0)	0	2.2 (2.0)	2.2 (2.0)	0	90.1			100		
(−)(11*S*, 2′*R*) RO 13-7225	Juvenile	50	0/5	0.6 (0.5)	25.2 (15.2)	25.8 (15.3)	13.6 (7.8)	12.2 (7.5)	19.9	12.90	<0.001	16.4	12.20	<0.001
		100	0/5	0.6 (0.5)	2.4 (1.7)	3.0 (1.4)	1.6 (0.9)	1.4 (0.5)	90.7			90.4		
		200	0/5	2.4 (1.5)	0.8 (1.8)	3.2 (2.9)	2.2 (1.6)	1.0 (1.4)	90.1			93.2		
		400	1/5	0.8 (0.4)	1.8 (2.1)	2.6 (2.3)	1.8 (1.5)	0.8 (0.8)	91.9			94.5		
	Adult	50	0/5	5.2 (4.6)	22.2 (8.6)	29.4 (7.9)	15.2 (3.6)	12.2 (3.9)	8.7	13.19	<0.001	16.4	13.88	<0.001
		100	0/5	10.4 (2.1)	3.4 (2.3)	13.8 (3.4)	11.4 (2.1)	2.4 (1.7)	57.1			83.6		
		200	0/5	6.8 (3.7)	1.4 (2.6)	8.2 (5.8)	7.8 (5.1)	0.4 (0.9)	74.5			97.3		
		400	0/5	3.8 (1.8)	0.2 (0.4)	4.0 (2.0)	4.0 (2.0)	0	87.6			100		

Worm burden is stratified by sex and worm distribution.

KW, Kruskal Wallis test; SD, standard deviation.

a Two mice died at days 14 and 17 post-treatment.

Mefloquine (+)(11*R*,2′*S*) yielded total and female worm burden reductions of 64.0% to 100% in mice harboring 21-day-old juvenile and 49-day-old adult *S. mansoni* at a single dose of 100 mg/kg and above. At the lowest dose investigated, 50 mg/kg of mefloquine (+)(11*R*,2′*S*), we still observed moderate total and female worm burden reductions (36.0–49.1%).

### Dose-response relationships of mefloquine against juvenile and adult *S. japonicum*

Table 6 summarizes the dose-response relationship of mefloquine against 14-day-old juvenile and 35-day-old adult *S. japonicum* in the mouse model. At the lowest dose investigated (25 mg/kg), total and female worm burden reductions of 16.9% and 10.9%, respectively, were observed against juvenile *S. japonicum*. Slightly higher total and female worm burden reductions (48.9% and 43.6%, respectively) were found when the dose of mefloquine was doubled (50 mg/kg). Administration of single doses of 100 mg/kg to 400 mg/kg achieved high total and female worm burden reductions of 86.7% and 95.1%. There was a highly significant difference (*p*<0.001) in the medians of the total and female worm burden between treated (50–400 mg/kg) and untreated control mice that were infected with *S. japonicum* 14 days previously.

**Table 6:** Dose response relationship of mefloquine administered to mice harboring either a 14-day-old juvenile or a 35 day-old adult *S. japonicum* infection.

Stage of infection	Dosage (mg/kg)	No. of mice investigated	No. of mice cured	Mean number of worms (SD)			Total worm burden reduction (%)	KW	*p*-value	Female worm burden reduction (%)	KW	*p*-value
				Total	Males	Females						
Juvenile	–	8	–	22.5 (8.3)	12.4 (3.4)	10.1 (2.9)	–	12.19	<0.001	–	11.60	<0.001
	25	6	0	18.7 (6.0)	9.7 (3.5)	9.0 (2.5)	16.9			10.9		
	50	6	0	11.5 (6.4)	5.8 (3.2)	5.7 (3.3)	48.9			43.6		
	100	6	1	3.0 (2.8)	1.8 (1.7)	1.2 (1.2)	86.7			88.1		
	200	5	3	1.4 (2.2)	0.6 (0.9)	0.8 (1.3)	93.8			92.1		
	400	6	4	1.2 (2.0)	0.7 (1.2)	0.5 (0.8)	94.7			95.1		
Adult	–	5	–	27.2 (4.4)	16.2 (4.4)	11.4 (1.3)	–	10.86	0.001	–	9.40	0.002
	25	4	0	20.0 (5.4)	10.5 (3.1)	9.5 (2.4)	26.4			16.7		
	50	8	0	16.4 (8.6)	8.5 (4.5)	7.9 (4.1)	39.7			30.7		
	100	8	0	7.9 (5.4)	4.9 (2.7)	3.0 (3.1)	70.9			73.7		
	200	8	0	3.4 (2.7)	3.3 (2.5)	0.1 (0.4)	87.5			99.1		
	400	8	4	1.3 (1.4)	1.3 (1.4)	0	95.2			100.0		

Worm burden is stratified by sex and worm distribution.

KW, Kruskal Wallis test; SD, standard deviation.

Administration of mefloquine at a single-dose of either 25 mg/kg or 50 mg/kg to mice infected with adult *S. japonicum* resulted in total and female worm burden reductions ranging between 16.7% and 39.7%. A higher dose of mefloquine (100 mg/kg) given to mice harboring adult *S. japonicum* resulted in total and female worm burden reductions of 70.9% and 73.7%, respectively. Total and female worm burden reductions of 87.5% to 100% were obtained with mefloquine at 200 mg/kg and 400 mg/kg in the adult infection model. Total worm counts recovered from treated (25–400 mg/kg) mice were significantly different from non-treated control mice infected with adult *S. japonicum* (KW = 10.86, *p* = 0.001 and KW = 9.40, *p* = 0.002, respectively).

### Stage-specific susceptibility of *S. japonicum*

A single oral dose of 400 mg/kg mefloquine was used to investigate the stage-specific susceptibility of *S. japonicum* because this dose showed the highest activities against juvenile and adult stages of *S. japonicum* in our previous experiments. Mefloquine achieved high total and female worm burden reductions ranging between 77.3% and 100% when administered to mice infected with *S. japonicum* for 3 to 35 days (Table 7). Lower total and female worm burden reductions (26.3–49.2%) were observed when mefloquine was administered 1 or 3 days before infection (days 1 and 3 prior to infection) or shortly after infection (3 hours post-infection). The difference in total and female worm burden reductions between treated and untreated control mice was highly significant regardless of the point in time mefloquine was administered (both *p*<0.001).

**Table 7:** Stage specificity of a single 400 mg/kg oral dose of mefloquine administered to mice infected with *S. japonicum*, stratified by sex.

Drug administration		No. of mice investigated	No. of mice cured	Mean number of worms (SD)			Total worm burden reduction (%)	KW	*p*-value	Female worm burden reduction (%)	KW	*p*-value
				Total	Males	Females						
	Control	5	–	27.2 (4.4)	16.2 (4.4)	11.4 (1.3)	-	12.51	<0.001	–	12.59	<0.001
Pre-infection	Day -2	5	0	16.8 (5.3)	9.8 (3.3)	7.0 (2.7)	38.3			38.6		
	Day -1	5	0	18.0 (3.4)	10.4 (3.1)	8.4 (1.5)	33.4			26.3		
	Day 0	4	0	15.5 (5.4)	9.8 (3.5)	5.8 (2.2)	43.0			49.2		
Post-infection	Day 3	4	0	4.8 (1.9)	2.5 (1.0)	2.3 (1.0)	82.4			79.8		
	Day 7	5	0	3.4 (1.3)	2.0 (0.7)	1.4 (0.9)	87.5			87.7		
	Day 14	4	3	0.5 (1.0)	0.3 (0.5)	0.3 (0.5)	98.2			97.4		
	Day 21	5	0	6.2 (5.0)	3.4 (2.6)	2.8 (3.1)	77.3			75.4		
	Day 28	5	0	5.8 (2.6)	4.6 (2.6)	1.2 (1.3)	78.8			89.5		
	Day 35	5	1	2.0 (1.2)	2.0 (1.2)	0	92.6			100.0		

KW, Kruskal-Wallis test; SD, standard deviation.

### Hepatic shift

In Table 8 and Table S3 we summarize the distribution of adult schistosomes in the liver and mesenteric veins on days 1, 3, 7, and 14 following mefloquine administration in *S. mansoni*- and *S. japonicum*-infected mice. The hepatic shift commenced one day post-treatment, and nearly all worms had shifted to the liver on day 3 post-treatment in both infection models. The worm size was smaller, and the majority of worm pairs were separated. On day 14, the majority of the worms had been eliminated.

**Table 8:** Hepatic shift test following a single 400-mg/kg oral dose of mefloquine administered to mice infected with *S. mansoni* and *S. japonicum*.

Schistosome species	Day of analysis post-treatment	No. of mice investigated	Number of worms liver		Number of worms mesenteric veins		Total worm burden
			Mean (SD)	%	Mean (SD)	%	Mean (SD)
*S. mansoni*	Control	10	4.3 (1.9)	12.0	31.6 (6.4)	88.0	35.9 (6.3)
	Day 1	5	16.2 (1.6)	37.9	26.6 (6.8)	62.1	42.8 (6.5)
	Day 3	5	37.0 (4.1)	96.4	1.4 (2.2)	3.6	38.4 (4.2)
	Day 7	4	9.3 (4.1)	100	0	0	9.3 (4.1)
	Day 14	5	5.8 (2.8)	100	0	0	5.8 (2.8)
*S. japonicum*	Control	5	0.2 (0.4)	1.2	16.2 (2.5)	98.8	16.4 (2.3)
	Day 1	5	11.5 (7.9)	47.3	12.8 (11.1)	52.7	24.3 (6.2)
	Day 3	4	18.6 (4.0)	100	0	0	18.6 (4.0)
	Day 7	5	12.2 (1.9)	95.3	0.6 (0.9)	4.7	12.8 (2.4)
	Day 14	5	5.6 (2.2)	87.5	0.8 (1.8)	12.5	6.4 (2.6)

SD, standard deviation.

## Discussion

We report promising antischistosomal properties of mefloquine, a marketed drug for prophylaxis and treatment of malaria. Oral administration of a single dose of mefloquine (400 mg/kg) to mice infected with either juvenile or adult stages of *S. mansoni* and *S. japonicum,* two of the three most important schistosome species [5], resulted in very high or complete total and female worm burden reductions. Interestingly, a recent study, which used a lower dose of mefloquine (150 mg/kg), reported no effect on the worm burden, but a reduction in egg fecundity in the first three developmental stages of *S. mansoni* in the murine model [25]. The discrepancy of the activity of mefloquine, when administered at doses of 100 mg/kg or 200 mg/kg to mice infected with adult *S. mansoni* observed in our study (worm burden reduction of 45% and 72%, respectively) and the previous investigation (no worm burden reduction) [25] remains to be elucidated. Previous research has shown that mefloquine also exhibits a broad spectrum of antimicrobial activity [26], as well as activity against larval and adult stages of *Brugia patei* and *B. malayi in vitro* [27].

If our results can be confirmed in proof-of-concept studies in humans, initially with current antimalarial dosages, it is conceivable that mefloquine can play a role in public health because the drug is widely and effectively used in malaria-endemic settings [28],[29], and because of the fact that malaria and schistosomiasis co-exist over large parts of sub-Saharan Africa and elsewhere [22]. The highest activities in *S. mansoni-* and *S. japonicum*-infected mice were observed when mefloquine was given at a single oral dose of 200–400 mg/kg, which correspond to 16–31 mg/kg in humans (dose calculator: http://www.fda.gov/cder/cancer/animalframe.htm). At present, the recommended dosage of mefloquine is 25 mg/kg when used in human treatment of malaria.

**Figure 2:**
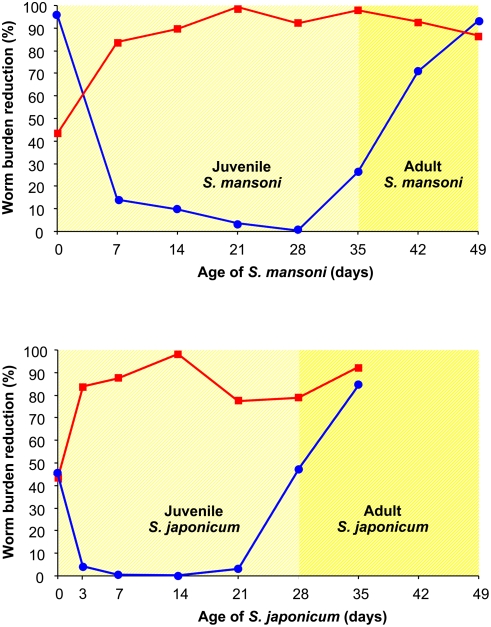
Stage-specific susceptibility of mefloquine compared to praziquantel in the *S. mansoni-* and *S. japonicum*-mouse models. Red squares: mefloquine 400 mg/kg, blue circles: praziquantel 400 mg/kg. The stage-specific susceptibilities of praziquantel and mefloquine on *S. mansoni* have been established in the laboratories of the Swiss Tropical Institute (Basel, Switzerland). The efficacy of praziquantel against *S. japonicum* has been reported by Yue et al. [51] and that of mefloquine has been established at the laboratories of the National Institute of Parasitic Diseases (Shanghai, China).

In contrast to other recently portrayed schistosomicides such as the oxadiazoles [10] or the cysteine protease inhibitor K11777 [14], which thus far have only been tested intraperitoneally and in multiple doses, mefloquine at a single oral dose resulted in high worm burden reductions. Moreover, the consistently high worm burden reductions observed against all development stages of the schistosome worms in the rodent model seems to be an advantage of mefloquine over praziquantel; the latter only displaying high activity against very young stages (skin penetration) and adult schistosomes [30],[31]. Actually, the minor activity of praziquantel against juvenile (2- to 3-week-old) schistosomes is believed to be a key factor explaining observed treatment ‘failures’ in areas highly endemic for schistosomiasis and that require frequent retreatments [11],[32],[33]. For comparison, the stage-specific susceptibility of praziquantel and mefloquine are juxtaposed in Figure 2. It is evident that mefloquine exceeds benchmark criteria set forth by the World Health Organization (WHO) for highly active lead compounds (defined as worm burden reduction of >80% in the adult *S. mansoni*-mouse model following five consecutive doses given intraperitoneally or subcutaneously) [34]. We approached or exceeded this benchmark level with a single dose of 400 mg/kg given orally to mice infected with either adult or juvenile stages of *S. mansoni* and *S. japonicum*.

Single oral doses of the antimalarials amodiaquine, atovaquone, sulfadoxine, sulfamethoxypyrazine, pyronaridine and pyrimethamine showed no activity in the *S. mansoni*-mouse model. A single oral dose of chloroquine also had no activity in the *S. mansoni*-mouse model, while previous work documented antischistosomal properties when the drug was given as multiple intraperitoneal doses [35]. On the other hand, quinine and halofantrine, which are structurally related to mefloquine, exhibited promising antischistosomal properties in the mouse (total worm burden reductions >50% following a single-dose oral regimen). The lack of antischistosomal activity of oral lumefantrine, which also belongs to the class of aminoalcohols, might be explained by the low oral bioavailability of this drug [36]. Further studies with intraperitoneal lumefantrine in *S. mansoni*-infected mice are ongoing in our laboratories. Interestingly, oxamniquine, a drug that has been widely and effectively used for the treatment and control of schistosomiasis mansoni, particularly in Brazil where more than 12 million people have been administered this drug [37], also contains an aminoalcohol functionality [38].

Importantly, previous investigations on the morphology of schistosomes recovered from host animals after administration of praziquantel [39] and observations made with schistosomes collected from mefloquine-treated mice point to different mechanisms of actions. Results obtained from preliminary morphological investigations (no data shown) indicate that mefloquine exerts a rapid action on schistosomes, resulting in marked alterations of the digestive tract and the reproductive system of the worms. The detailed mechanism of action of mefloquine and related aminoalcohols on schistosomes remains to be investigated. Still today, the exact mechanism of action of mefloquine on *Plasmodium* is not known, though interference with hemoglobin digestion seems to play a role [40]. It was demonstrated that the antimalarial chloroquine inhibits the formation of hemozoin, a heme detoxification aggregate in *S. mansoni* female homogenates [35], hence future studies should elucidate whether mefloquine also targets hemozoin formation. It is interesting to note that adult female *S. mansoni* were more affected by mefloquine at doses of 100–400 mg/kg than male adult *S. mansoni*. This phenomenon was not observed when treating juvenile stages of either *S. mansoni* or *S. japonicum*. Differences in drug susceptibility between male and female *S. mansoni* have been reported previously, e.g., following hycanthone treatment [41] and point to a sex-specific interference of the drug with the target, or different drug targets.

Interestingly, moderate worm burden reductions were found when a single dose of mefloquine was given one or two days before or three hours after infection of mice with either *S. mansoni* or *S. japonicum*. Although mefloquine has a long half-life of 6.5 to 22.7 days in healthy volunteers [42], the half-life of this drug in mice is much shorter, namely 17 hours [43]. Further studies should be launched to clarify whether active metabolites, the significant enterohepatic recirculation of mefloquine found in rodents [44] or a treatment induced alteration of the immune response, which has, for example, been described for praziquantel, [45] may be a contributing factor to the efficacy and be partly responsible for the moderate worm burden reductions recorded when mefloquine is given pre-infection. Pro-inflammatory effects of mefloquine have been described in both *S. mansoni*-infected and non-infected mice [25].

Mefloquine is generally well tolerated by adults and children. However, there is evidence that mefloquine may result in harmful events in the gastrointestinal and central nervous systems. Observed adverse events include insomnia, nausea, vomiting, diarrhea, headache, dizziness, rash, pruritus, and abdominal pain. Severe neuropsychiatric symptoms as seizures and hallucinations are rare (occurring in 1 per 10,000 patients) [46]. Adverse events seem to be dose-dependent, and it was suggested that doses greater than 15 mg/kg should be divided [42]. To date, the neurotoxicity of mefloquine cannot be explained, and it remains to be elucidated whether there is steroselectivity in the neurotoxic potencies of the enantiomers. However, the brain penetration of the (+) enantiomer was found to be much higher than that of the (−) enantiomer in two post-mortem human cerebral biopsies [47]. We have demonstrated that both enantiomers and the racemic hydrochloride possess a similar activity against juvenile and adult *S. mansoni.* Interestingly, this finding contrasts with the activity of mefloquine in *Plasmodium yoelii*-infected mice, where the racemic hydrochloride of mefloquine showed a two-fold to three-fold higher activity when compared to the enantiomers, which had similar activity to each other [48].

In conclusion, we have documented promising *in vivo* antischistosomal efficacy of the antimalarial drug mefloquine. New studies have been launched with an aim to elucidate the effect of mefloquine on the third major schistosome species, i.e., *S. haematobium,* and a number of the biologically-related trematodes such as *Clonorchis sinensis* and *Opisthorchis viverrini*. Additionally, *in vitro* studies and an evaluation of the potential of praziquantel-mefloquine combination therapy have been initiated. Finally, a proof-of-concept study will be launched in an African setting, where malaria and schistosomiasis co-exist, similar to our preceding work with the artemisinins [49],[50]. A word of caution should be mentioned here, as the malaria community has argued that antimalarial drugs should not be used against schistosomiasis because of concern that this strategy might select for *Plasmodium*-resistant parasites. However, millions of people have been, or will be, treated with mefloquine and mefloquine-artesunate combinations in areas where both malaria and schistosomiasis co-exist [22]. Hence, the potential ancillary benefit of the antimalarial drug mefloquine should be investigated against schistosomiasis.

## Supporting Information

Alternative Language Abstract S1 Translation of the Abstract into Chinese by Shu-Hua Xiao
(0.08 MB PDF)

Alternative Language Abstract S2 Translation of the Abstract into German by Jennifer Keiser
(0.02 MB PDF)

Table S1 Dose-response relationship of mefloquine administered to mice harboring a 21-day-old juvenile and a 49-day-old adult *S. mansoni* infection.
(0.01 MB PDF)

Table S2 Stage-specificity of a single 400 mg/kg oral dose mefloquine administered to mice infected with *S. mansoni*, stratified by sex and worm distribution.
(0.02 MB PDF)

Table S3 Hepatic shift test following a single 400-mg/kg oral dose of mefloquine administered to mice infected with *S. mansoni*.
(0.01 MB PDF)
