# Supplementary material for: Mefloquine—An Aminoalcohol with Promising Antischistosomal Properties in Mice
Source: PLoS Negl Trop Dis. 2009 Jan 6;3(1):e350. doi: 10.1371/journal.pntd.0000350 (PMC2600813; doi:10.1371/journal.pntd.0000350)
Supplement: Alternative Language Abstract S1 — Translation of the Abstract into Chinese by Shu-Hua Xiao (0.08 MB PDF) [file pntd.0000350.s001.pdf]

**甲氟喹，一种具有抗小鼠血吸虫作用的氨基醇化合物**

Translation of the Abstract into Chinese by Xiao Shu-Hua


**摘要**

**背景**：一种被忽视的、使贫穷恶化的热带病，即血吸虫病的防治仅依赖于吡喹酮一种药物。大规模应用吡喹酮有可能挑选出对吡喹酮具有抗性的血吸虫，因此需发展新的抗血吸虫药物。本文报道抗疟药甲氟喹对小鼠模型具有很好的抗血吸虫作用。

**方法学/主要发现**：感染曼氏血吸虫和日本血吸虫成虫的小鼠口服单剂甲氟喹（200 mg/kg 和 400 mg/kg），对总的和雌虫负荷的减少高达 72.3%-100%。重要的是，在小鼠模型中亦观察到甲氟喹治疗对不同发育期的曼氏血吸虫和日本血吸虫童虫具有很高的虫负荷的减少。甲氟喹的 2 种立体异构体对感染曼氏血吸虫成虫或童虫的小鼠均有很高的和相仿的总的和雌虫负荷的减少。

**结论/意义**：我们的发现有望在氨基乙醇化合物作用的基础上发展新的抗血吸虫药物。进一步阐明甲氟喹抗血吸虫的可能作用方式及研究其对埃及血吸虫和食源性吸虫疗效的体外和体内试验业已开展。我们认为，广泛和有效地应用于治疗疟疾的甲氟喹将在疟疾和血吸虫病共存的流行地区，对血吸虫病的防治产生影响。
