# Supplementary material for: Mefloquine—An Aminoalcohol with Promising Antischistosomal Properties in Mice
Source: PLoS Negl Trop Dis. 2009 Jan 6;3(1):e350. doi: 10.1371/journal.pntd.0000350 (PMC2600813; doi:10.1371/journal.pntd.0000350)
Supplement: Alternative Language Abstract S2 — Translation of the Abstract into German by Jennifer Keiser (0.02 MB PDF) [file pntd.0000350.s002.pdf]

Mefloquin: ein Aminoalkohol mit vielversprechender Aktivität gegen Bilharziose im Maus Model

Translation of the Abstract into German by Jennifer Keiser

Zusammenfassung

Hintergrund: Für die Behandlung und Kontrolle der Bilharziose, eine chronische parasitäre Krankheit welche auch die Armut beeinträchtigen kann, steht nur ein einziges Medikament zur Verfügung: das Praziquantel. Da Resistenzentwicklung eine grosse Gefahr ist, sollten neue Medikamente für die Behandlung der Bilharziose entwickelt werden. In dieser Arbeit berichten wir, dass das Malariamedikament Mefloquin im Mausmodell eine vielversprechende Wirkung gegen *Schistosoma mansoni* und *S. japonicum* aufgezeigt hat.

Methoden/wichtigste Ergebnisse: Mäuse, welche mit *S. mansoni* oder *S. japonicum* im akuten oder chronischen Stadium infiziert waren und mit einer Einfachdosis Mefloquine (200 und 400 mg/kg) behandelt wurden, zeigten im Vergleich mit unbehandelten Mäusen eine deutliche Reduktion der Wurmbürde auf (72.3-100%). Mefloquine enantiomere resultierten ebenfalls in hohen Reduktionen der Wurmbürde nach Applikation von S. mansoni-infizierten Mäusen, sowohl im akuten wie auch im chronischen Stadium.

Schlussfolgerungen/Bedeutung: Eine Reihe von Studien sind nötig, um die schistosomiziden Eigenschaften des Mefloquins näher charakterisieren zu können. Da das Mefloquin in Gebieten eingesetzt wird, in welchen sowohl die Malaria als auch die Bilharziose ko-endemisch sind, könnten unser Ergebenisse für das öffentliche Gesundheitswesen von grosser Bedeutung sein.
