# Supplementary material for: Mefloquine—An Aminoalcohol with Promising Antischistosomal Properties in Mice
Source: PLoS Negl Trop Dis. 2009 Jan 6;3(1):e350. doi: 10.1371/journal.pntd.0000350 (PMC2600813; doi:10.1371/journal.pntd.0000350)
Supplement: Table S1 — Dose-response relationship of mefloquine administered to mice harboring a 21-day-old juvenile and a 49-day-old adult S. mansoni infection. (0.01 MB PDF) [file pntd.0000350.s003.pdf]

Supporting Information Table 1. Dose-response relationship of mefloquine administered to mice harboring a 21-day-old juvenile and a 49-day-old adult *S. mansoni* infection. Worm burden is stratified by sex and worm distribution.

Stage of infection	Dosage (mg/kg)	No. of mice investigated	No. of mice cured	Mean number of worms (SD)		Total worm burden reduction (%)	KW	*P*-value	Female worm burden reduction (%)	KW	*P*-value
				**Total**	**Females**						
Juvenile	-	10	-	37.0 (6.4)	14.7 (3.2)	-			-		-
	25	5	0	37.0 (8.9)	12.8 (3.3)	0			12.9		
	50	5	0	16.0 (10.4)	6.8 (4.8)	56.8			53.8		
	100	5	0	6.0 (1.2)	2.2 (0.4)	83.8	12.9	0.0003	85.0	14.98	0.0001
	200	5	2	1.0 (1.2)	0.2 (0.4)	97.3			98.6		
	400	5	4	0.4 (0.9)	0.2 (0.4)	98.9			98.6		
Adult	-	10	-	41.6 (8.0)	18.5 (4.2)	-			-		
	25	5	0	44.3 (7.4)	20.0 (5.0)	0			0		
	50	5	0	20.6 (9.8)	8.4 (5.0)	50.5			54.6		
	100	5	0	9.6 (2.9)	2.0 (1.0)	76.9	12.8	0.0003	89.2	12.5	0.0004
	200	5	0	8.2 (3.7)	0.6 (0.5)	80.3			96.8		
	400	5	0	2.8 (1.3)	0	93.3			100		

KW, Kruskal Wallis test, SD, standard deviation
