# Supplementary material for: Mefloquine—An Aminoalcohol with Promising Antischistosomal Properties in Mice
Source: PLoS Negl Trop Dis. 2009 Jan 6;3(1):e350. doi: 10.1371/journal.pntd.0000350 (PMC2600813; doi:10.1371/journal.pntd.0000350)
Supplement: Table S2 — Stage-specificity of a single 400 mg/kg oral dose mefloquine administered to mice infected with S. mansoni, stratified by sex and worm distribution. (0.02 MB PDF) [file pntd.0000350.s004.pdf]

Supporting Information Table 2. Stage-specificity of a single 400 mg/kg oral dose mefloquine administered to mice infected with *S. mansoni*, stratified by sex and worm distribution.

Drug administration		No. of mice investigated	No. of mice cured	Mean number of worms (SD)			Total worm burden reduction (%)	KW	*P*-value	Female worm burden reduction (%)	KW	*P*-value
				Total	Males	Females						
Pre-infection	Control	10	-	44.0 (10.3)	23.9 (5.3)	20.1 (6.0)	-	36.45	<0.001	-	35.05	<0.001
	Day -2	5	0	29.6 (6.9)	16.2 (3.0)	13.4 (4.4)	32.7			33.3		
	Day -1	5	0	22.0 (4.7)	12.2 (3.7)	9.8 (2.2)	50.0			51.3		
Post-infection	Control	10	-	45.8 (16.4)	27.3 (9.1)	18.3 (7.5)	-			-		
	Day 7	5	0	7.8 (1.1)	4.8 (1.5)	3.0 (0.7)	82.3			85.1		
	Day 14	5	2	5.8 (7.8)	3.4 (4.2)	2.4 (3.6)	86.8			88.1		
	Day 21	5	2	0.6 (0.5)	0.6 (0.5)	0	98.7			100		
	Day 28	5[a]	0	6.7 (6.7)	6.3 (6.1)	0.3 (0.6)	85.4			98.4		
	Day 35	5	1	1.2 (0.8)	1.2 (0.8)	0	97.4			100		
	Day 42	5	0	4.0 (1.4)	4.0 (1.4)	0	91.3			100		
	Day 49	5[a]	0	9.0 (3.0)	9.0 (3.0)	0	80.4			100		

KW, Kruskal-Wallis test, SD Standard deviation
a One mouse died several days post-treatment
