# Supplementary material for: Mefloquine—An Aminoalcohol with Promising Antischistosomal Properties in Mice
Source: PLoS Negl Trop Dis. 2009 Jan 6;3(1):e350. doi: 10.1371/journal.pntd.0000350 (PMC2600813; doi:10.1371/journal.pntd.0000350)
Supplement: Table S3 — Hepatic shift test following a single 400-mg/kg oral dose of mefloquine administered to mice infected with S. mansoni. (0.01 MB PDF) [file pntd.0000350.s005.pdf]

Supporting Information Table 3. Hepatic shift test following a single 400-mg/kg oral dose of mefloquine administered to mice infected with *S. mansoni.*

Day of analysis post-treatment	No. of mice investigated	Number of worms liver		Number of worms mesenteric veins		Total worm burden
		Mean (SD)	%	Mean (SD)	%	Mean (SD)
Control	10	4.3 (1.9)	12.0	31.6 (6.4)	88.0	35.9 (6.9)
Day 1	5	16.2 (1.6)	37.9	26.6 (6.8)	62.1	42.8 (6.2)
Day 3	5	37.0 (4.1)	96.4	1.4 (2.2)	3.6	38.4 (4.2)
Day 7	5	9.3 (4.1)	100	0	0	9.3 (4.1)
Day 14	5	5.8 (2.8)	100	0	0	5.8 (2.8)
